# Correction: Volkova et al. Cyclodextrin’s Effect on Permeability and Partition of Nortriptyline Hydrochloride. *Pharmaceuticals* 2023, *16*, 1022

**DOI:** 10.3390/ph17010057

**Published:** 2023-12-29

**Authors:** Tatyana Volkova, Olga Simonova, German Perlovich

**Affiliations:** G.A. Krestov Institute of Solution Chemistry RAS, 153045 Ivanovo, Russia; ors@isc-ras.ru (O.S.); glp@isc-ras.ru (G.P.)

## Error in Figure

In the original publication [1], there was a mistake in Figure 2 as published. An error was made in the location of the diagrams in Figure 2a,b. The diagrams did not match the figure caption regarding the pH of the aqueous phase of the distribution system. The corrected Figure 2 appears below. The authors state that the scientific conclusions are unaffected. This correction was approved by the Academic Editor. The original publication has also been updated.

## Figures and Tables

**Figure 2:**
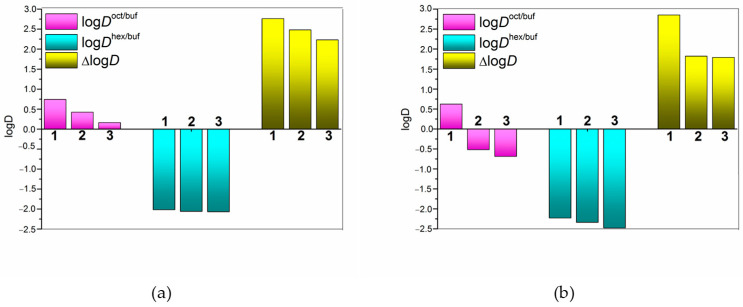
Distribution coefficients log$D_{app}^{oct/buf}$, log$D_{app}^{hex/buf}$, and ΔlogD parameter without cyclodextrins (1), with 0.0115 M of HP-β-CD (2), and with 0.0115 M of SBE-β-CD (3) in the aqueous phase for NTT•HCl at 37 °C: (**a**) pH 6.8, (**b**) pH 4.0 of the buffer phase.
