# Supplementary material for: Cyclodextrin’s Effect on Permeability and Partition of Nortriptyline Hydrochloride
Source: Pharmaceuticals (Basel). 2023 Jul 19;16(7):1022. doi: 10.3390/ph16071022 (PMC10386514; doi:10.3390/ph16071022)
Supplement: Supplementary file 1 [file pharmaceuticals-16-01022-s001.zip › pharmaceuticals-2493875-supplementary.pdf]

Supplementary Material

Cyclodextrin's Effect on Permeability and Partition of Nortriptyline Hydrochloride

Tatyana Volkova *, Olga Simonova and German Perlovich

G.A. Krestov Institute of Solution Chemistry RAS, 153045 Ivanovo, Russia; ors@isc-ras.ru (O.S.); glp@isc-ras.ru (G.P.)

* Correspondence: vtv@isc-ras.ru; Tel.: +7-(4932)351545

Table of Contents

Figure S1	Particles distribution plots.	2
Table S1	Molar concentrations (C_2) of NTT•HCl in the organic and aqueous phases of the studied distribution systems at 37 °C.	3
Table S2	Donor solution concentrations (*C*), flux (*J*), and permeability coefficients (P_{app}) of NTT•HCl through the PermePad barrier (PP) and cellulose membrane (RC) at 37 °C, pH 6.8 in the donor compartment; the standard deviations are given in parenthesis.	3

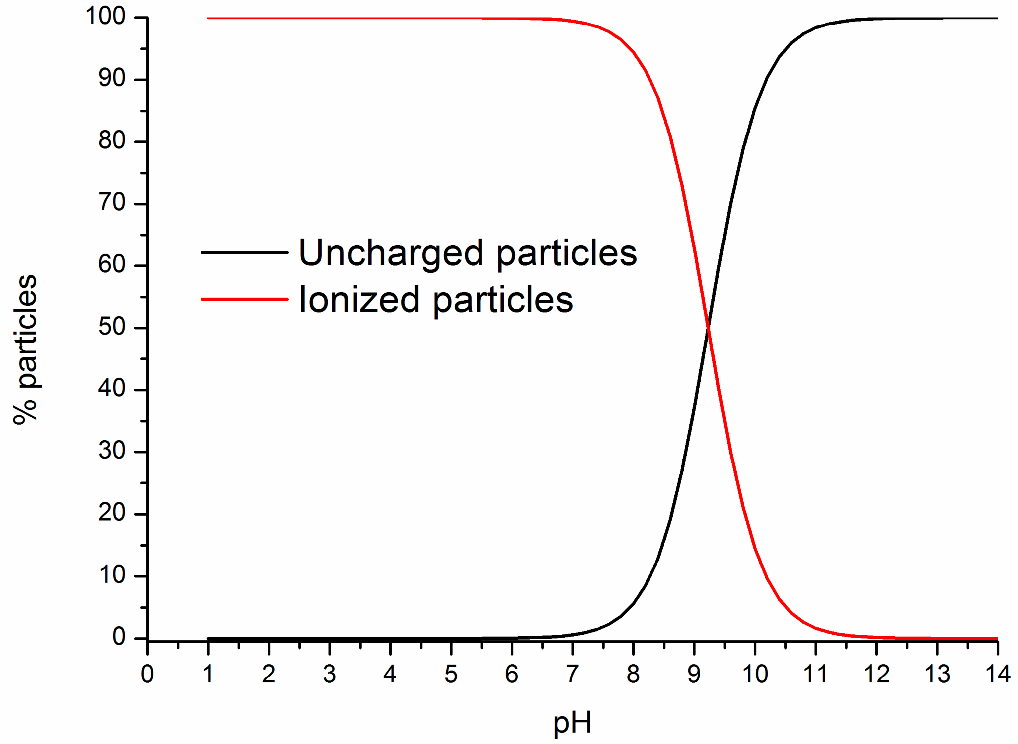

Figure S1. Particles distribution plots.

Table S1. Molar concentrations (C_2) of NTT•HCl in the organic and aqueous phases of the studied distribution systems at 37 °C.

System	$C_2^{Org/buf}$	$C_2^{buf/Org}$	$C_2^{Org/buf}\cdot10^4$	$C_2^{buf/Org}\cdot10^3$
	pH 6.8		pH 4.0	
	[1]1-octanol/buffer system			
Without CD	$1.82\cdot10^{-3}$	$3.29\cdot10^{-4}$	$1.58\cdot10^{-3}$	$3.79\cdot10^{-4}$
0.0115 M HP-β-CD	$1.38\cdot10^{-3}$	$5.29\cdot10^{-4}$	$4.99\cdot10^{-4}$	$1.67\cdot10^{-3}$
0.025 M HP-β-CD	$1.27\cdot10^{-3}$	$6.38\cdot10^{-4}$	$2.89\cdot10^{-4}$	$1.88\cdot10^{-3}$
0.035 M HP-β-CD	$1.11\cdot10^{-3}$	$7.90\cdot10^{-4}$	$1.99\cdot10^{-4}$	$1.97\cdot10^{-3}$
0.0115 M SBE-β-CD	$1.01\cdot10^{-3}$	$7.00\cdot10^{-4}$	$3.63\cdot10^{-4}$	$1.78\cdot10^{-3}$
0.025 M SBE-β-CD	$4.57\cdot10^{-4}$	$1.25\cdot10^{-3}$	$1.70\cdot10^{-4}$	$1.97\cdot10^{-3}$
0.035 M SBE-β-CD	$3.68\cdot10^{-4}$	$1.62\cdot10^{-3}$	$2.65\cdot10^{-5}$	$2.12\cdot10^{-3}$
	[2]n-hexane/buffer system			
Without CD	$1.11\cdot10^{-4}$	$1.95\cdot10^{-3}$	$6.58\cdot10^{-5}$	$1.86\cdot10^{-3}$
0.0115 M HP-β-CD	$1.13\cdot10^{-4}$	$2.16\cdot10^{-3}$	$5.46\cdot10^{-5}$	$1.97\cdot10^{-3}$
0.0115 M SBE-β-CD	$9.86\cdot10^{-5}$	$1.92\cdot10^{-3}$	$3.59\cdot10^{-5}$	$1.80\cdot10^{-3}$

[1]V(oct):V(buf)= 3:3;

[2]V(hex):V(buf)= 12:2.

The standard uncertainties are $u(t)$=0.2 °C. The relative standard uncertainties are $u_r(C_2^{oct/buf})$; $u_r(C_2^{buf/oct})$; $u_r(C_2^{hex/buf})$; and $u_r(C_2^{buf/hex})$=0.04.

Table S2. Donor solution concentrations (C), flux (J), and permeability coefficients (P_{app}) of NTT•HCl through the PermePad barrier (PP) and cellulose membrane (RC) at 37 °C, pH 6.8 in the donor compartment; the standard deviations are given in parenthesis.

System	$C\cdot10^3$ (M)	J (μM·cm^{-2}·s^{-1})	P_{app} (cm·s^{-1})	$C\cdot10^3$ (M)	J (μM·cm^{-2}·s^{-1})	P_{app} (cm·s^{-1})
		PP			RC	
			pH 6.8			
Buffer	1.42	$3.46\cdot10^{5}$	$2.44(0.07)\cdot10^{-5}$	2.11	$8.22\cdot10^{-5}$	$3.90(0.11)\cdot10^{-5}$
			HP-β-CD			
0.0115 M	2.20	$2.69\cdot10^{-6}$	$1.22(0.03)\cdot10^{-6}$	1.34	$2.03\cdot10^{5}$	$1.52(0.04)\cdot10^{-5}$
0.025 M	2.20	$2.58\cdot10^{-6}$	$1.17(0.03)\cdot10^{-6}$	1.66	$2.06\cdot10^{5}$	$1.25(0.03)\cdot10^{-5}$
0.035 M	2.23	$9.39\cdot10^{-7}$	$4.21(0.05)\cdot10^{-7}$	1.34	$1.47\cdot10^{5}$	$1.10(0.03)\cdot10^{-5}$
			SBE-β-CD			
0.0115 M	2.28	$1.48\cdot10^{-6}$	$6.50(0.15)\cdot10^{-7}$	2.27	$1.31\cdot10^{5}$	$5.77(0.20)\cdot10^{-6}$
0.025 M	2.22	$9.06\cdot10^{-7}$	$4.09(0.08)\cdot10^{-7}$	1.68	$8.43\cdot10^{-6}$	$5.00(0.14)\cdot10^{-6}$
0.035 M	2.36	$7.20\cdot10^{-7}$	$3.05(0.07)\cdot10^{-7}$	2.36	$1.03\cdot10^{-5}$	$4.36(0.09)\cdot10^{-6}$
